# Organ-Specific Immune-Related Adverse Events Associated With Immune Checkpoint Inhibitor Monotherapy Versus Combination Therapy in Cancer: A Meta-Analysis of Randomized Controlled Trials

**DOI:** 10.3389/fphar.2019.01671

**Published:** 2020-01-30

**Authors:** Lijun Da, Yuanjun Teng, Na Wang, Karen Zaguirre, Yating Liu, Yali Qi, Feixue Song

**Affiliations:** ^1^ Department of Oncology, Lanzhou University Second Hospital, Lanzhou University, Lanzhou City, China; ^2^ Department of Orthopaedics, Lanzhou University Second Hospital, Lanzhou University, Lanzhou City, China; ^3^ Deparment of Surgery, St. Luke’s Medical Center, Quezon City, Philippines

**Keywords:** immune checkpoint inhibitor, combination immunotherapy, organ specific, adverse events, meta-analysis

## Abstract

**Background:**

Although combination therapy with immune checkpoint inhibitors (ICIs) provides a promising efficacy in multiple cancers, their use is facing challenges for a high incidence of adverse effects. This meta-analysis was conducted to compare the risks of organ-specific immune-related adverse events (IRAEs) associated with ICI monotherapy versus combination therapy among cancer patients.

**Methods:**

Electronic databases were systematically searched to include eligible randomized controlled trials (RCTs). Any-grade and 3-5 grade IRAEs (colitis, pneumonitis, hepatitis, hypothyroidism, hyperthyroidism, and hypophysitis) were extracted for meta-analysis. Two reviewers independently assessed the methodological quality. The RevMan 5.3.5 software was used for meta-analysis.

**Results:**

A total of 10 studies involving 8 RCTs with 2716 patients were included in this study. The most common any-grade adverse event was colitis (14.5%), followed by hypothyroidism (13.8%), hepatitis (10.4%), hypophysitis (10.0%), hyperthyroidism (9.3%), and pneumonitis (4.6%). Meta-analysis showed that ICI combination therapy significantly increased the risks of any-grade IRAEs in colitis [relative risk (RR), 3.56; 95% confidence interval (CI), 1.56–8.12; *p* < 0.05], pneumonitis (RR, 2.31; 95% CI, 1.54–3.45; *p* < 0.05), hepatitis (RR, 2.54; 95% CI, 1.65–3.91; *p* < 0.05), hypothyroidism (RR, 2.17; 95% CI, 1.71–2.76; *p* < 0.05), hyperthyroidism (RR, 3.13; 95% CI, 2.08–4.70; *p* < 0.05), and hypophysitis (RR, 3.54; 95% CI, 2.07–6.07; *p* < 0.05) compared with ICI monotherapy, as well as 3-5 grade IRAEs in colitis (RR, 2.50; 95% CI, 1.62–3.86; *p* < 0.05), pneumonitis (RR, 1.99; 95% CI, 1.00–3.93; *p* = 0.05), and hepatitis (RR, 2.70; 95% CI, 1.29–5.63; *p* < 0.05).

**Conclusions:**

This meta-analysis demonstrated that, compared with ICI monotherapy, patients receiving ICI combination therapy significantly increased organ-specific IRAEs in colitis, hypothyroidism, hepatitis, hypophysitis, hyperthyroidism, and pneumonitis. The incidence and severity of organ-specific IRAEs were drug and dose independent.

## Introduction

Immune checkpoint inhibitors (ICI) have shown remarkable efficacy in the therapy of multiple cancers, such as non-small cell lung carcinoma, renal cell carcinoma, head and neck squamous cell carcinoma, and melanoma (Mellman et al., 2011; Luke et al., 2017; Proto et al., 2019). The most widely used ICIs include cytotoxic T lymphocyte-associated protein 4 (CTLA4) and programmed death-1/ligand-1 (PD-1/PD-L1) inhibitors. These inhibitors block the agent interaction with the key immune regulatory pathways, thereby increasing the antitumor immunity (Johnson et al., 2017). Representative drugs of CTLA-4 (ipilimumab), PD-1 (nivolumab, pembrolizumab), and PD-L1 (avelumab, atezolizumab, and durvalumab) have been approved by the Food and Drug Administration (FDA) for malignant tumors.

In recent years, the combined use of PD-1 and CTLA-4 inhibitors has attracted increasing attention for the promising efficacy in the treatment of advanced melanoma, lung cancer, and sarcoma (Larkin et al., 2015; D’angelo et al., 2018; Hellmann et al., 2018a; Hellmann et al., 2018b). In patients with advanced melanoma, combination therapy with nivolumab and ipilimumab had significantly improved clinical outcomes with prolonged progression-free survival (PFS) and higher objective response rate (ORR) compared with ipilimumab alone (Postow et al., 2015; Hodi et al., 2016). Four clinical trials (CheckMate 012/032/227/568) demonstrated a durable response associated with ICI combination therapy among patients with lung cancer (Antonia et al., 2016; Hellmann et al., 2017; Hellmann et al., 2018b; Ready et al., 2019). Although ICI combination has become a significant breakthrough in cancer therapeutics, their use was associated with toxic effects resulting from unbalanced activation of the immune system. To distinguish from other treatment-related side effects, these toxic effects caused by immune activation were specifically termed as immune-related adverse events (IRAEs) (Postow et al., 2018).

IRAEs may occur in almost any organ, such as the colon, lungs, liver, muscle, and thyroid. According to the published study (Baxi et al., 2018), IRAEs were classified into three categories: organ-specific IRAEs (colitis, hepatitis, pnemonitis, etc.), general IRAEs (fatigue, diarrhea, and rash) and musculoskeletal IRAEs (arthritis, arthralgia, back pain, etc.). They demonstrated that the general adverse events are more prevalent, but the organ-specific IRAEs are more clinically important. Yang et al. (Yang et al., 2019) also suggested that oncologists should focus on the organ-specific IRAEs, which are more meaningful in clinical practice. Therefore, the organ-specific adverse event has been a new challenge in the treatment of cancers (Baxi et al., 2018; Martins et al., 2019).

Currently, although several meta-analysis have evaluated the efficacy and safety of ICIs (Wang et al., 2017; Barroso-Sousa et al., 2018; Ma et al., 2018; Wang et al., 2018; You et al., 2018), most studies included chemotherapy as the control group for the analysis, and few studies specifically assessed the safety of ICIs. A published meta-analysis by Wang et al. in 2018 reported fatal toxic effects associated with ICIs. They demonstrated that the organ-specific IRAEs were the most common causes for death: colitis for CTLA-4 (70%, 135/193 deaths), pneumonitis (35%, 115/333 deaths) for PD-1 or PD-L1 inhibitors, and colitis (37%, 32/87) for the combination PD-1 and CTLA-4 (Baxi et al., 2018). However, they failed to provide the detailed data about the incidences of low-grade and high-grade adverse events.

A comprehensive understanding of the epidemiology of the organ-specific IRAEs is essential for clinicians to balance the benefits and risks of ICI combination during cancer treatment (Martins et al., 2019). Therefore, we conducted this meta-analysis based on randomized controlled trials (RCTs) aiming to compare the organ-specific IRAEs of ICI monotherapy versus combination therapy among cancer patients.

## Materials and Methods

This study was performed based on the preferred reporting items for systematic reviews and meta-analyses (PRISMA) statement.

### Inclusion and Exclusion Criteria

The following inclusion criteria were used in this study: (1). types of included studies: randomized controlled trials (RCTs); (2). types of participants: patients over 18 years of age diagnosed with malignancies regardless of region, racial, and gender; (3). interventions: patients received the intervention treatment of either ICI monotherapy or combined therapy with CTLA-4/PD-1/PD-L1 antibodies; (4). types of outcomes: colitis, pneumonitis, hepatitis, hypothyroidism, hyperthyroidism, and hypophysitis. The severity of adverse events were graded according to the National Cancer Institute Common Terminology Criteria for Adverse Events version (CTCAE) 4.0, and grade ≥3 were evaluated as high grade or severe grade.

The exclusion criteria were: (1). types of studies: ongoing trials, quasi-RCT, non-RCT, reviews, commentaries, conference paper, and quality of life studies; (2) interventions: patients treated with placebo, chemotherapy, or chemotherapy plus immunotherapy.

### Data Sources and Searches

A literature search was conducted to identify RCTs comparing ICI monotherapy versus combination therapy among cancer patients. Without the restriction on language and publication status, the databases of MEDLINE, EMBASE, and Cochrane databases and ISI Web of Knowledge were searched to determine potentially eligible studies up to May 30, 2019. The following search terms were used: CTLA-4, ipilimumab, tremelimumab, PD-1, nivolumab, pembrolizumab, PDL-1, atezolizumab, avelumab, durvalumab, and checkpoint inhibitors. Additionally, the reference lists of identified studies and Google scholar were checked for other potentially eligible trials.

### Data Collection and Quality Assessment

Two blinded authors (Da and Teng) independently extracted data according to a standardized extraction form. Any discrepancy was resolved by discussion with a third author. If insufficient data was reported, efforts were made to contact the authors for the additional information. The methodological quality of the eligible studies was evaluated using the following items recommended by the Cochrane Collaboration: randomization, allocation concealment blinding of participant, blinding of outcome assessors, incomplete outcome data, selective reporting, and other bias (Higgins et al., 2011).

### Statistical Analysis

Meta-analysis was conducted using the software Review Manager 5.3.5. Risk ratio (RR) and 95% confidence interval (95% CI) were calculated to estimate the event rates for dichotomous outcomes. Heterogeneity was tested using *I* ^2^ index and the Cochran Q statistic (*I* ^2^ > 50% indicating significant heterogeneity, and *I* ^2^ ≤ 50% indicating no significant heterogeneity). If no heterogeneity (*I* ^2^ ≤ 50%) was presented in the meta-analysis, a fixed-effect model was used to estimate the pooled odds ratio and 95% confidence interval, otherwise, a random-effect model was used. Subgroup analyses were performed to explore the sources of heterogeneity according to the different tumors types.

## Results

**Figure 1:**
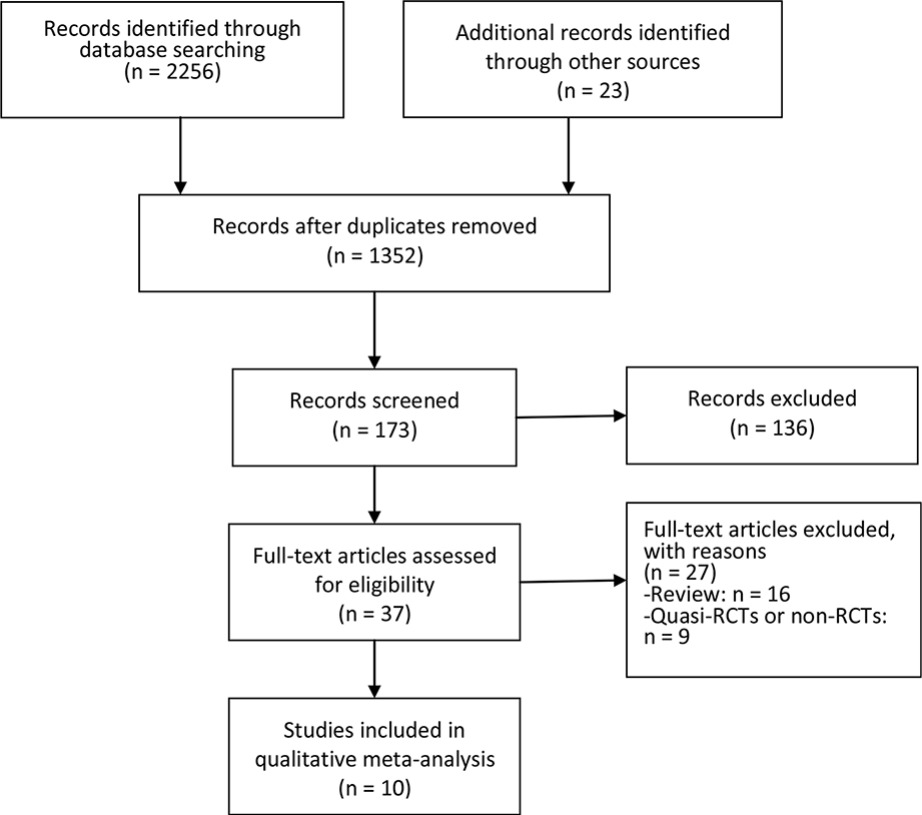
PRISMA flowchart of literature screening.

**Figure 1** showed the flow chart of literature screening. A total of 2,279 records were yielded in the initial search from the database. After removing duplicates, 1,352 studies were assessed for abstract and full-text review. Finally, 10 studies involving eight RCTs were included in this meta-analysis (Larkin et al., 2015; Postow et al., 2015; Antonia et al., 2016; Hodi et al., 2016; Wolchok et al., 2017; D’angelo et al., 2018; Hellmann et al., 2018b; Long et al., 2018; Omuro et al., 2018; Sharma et al., 2019).

### The Characteristics and Quality Assessment of Included RCTs

The detailed characteristics of included RCTs were shown in **Table 1**. A total of 2,716 patients (monotherapy group, 1,315; combination group, 1,401) were included in the analysis. In the combination group, all the patients received intervention with nivolumab and ipilimumab. Three studies compared the efficacy of two different doses of drug combinations: nivolumab 3 mg/kg plus ipilimumab 1 mg/kg (N3I1), or nivolumab 1 mg/kg plus ipilimumab 3 mg/kg (N1I3) (Antonia et al., 2016; Omuro et al., 2018; Sharma et al., 2019). In the monotherapy group, patients received intervention with either ipilimumab (Larkin et al., 2015; Postow et al., 2015; Hodi et al., 2016; Wolchok et al., 2017) or nivolumab alone (Larkin et al., 2015; Antonia et al., 2016; Wolchok et al., 2017; D’angelo et al., 2018; Hellmann et al., 2018b; Long et al., 2018; Omuro et al., 2018; Sharma et al., 2019). The included RCTs involved five kinds of tumors: lung cancer in two studies (Antonia et al., 2016; Hellmann et al., 2018b), melanoma in three studies (Larkin et al., 2015; Postow et al., 2015; Hodi et al., 2016; Wolchok et al., 2017; Long et al., 2018), metastatic sarcoma in one study (D’angelo et al., 2018), urothelial carcinoma (Sharma et al., 2019), and recurrent glioblastoma in one study (Omuro et al., 2018). All the included RCTs used the CTCAE 4.0 to evaluate the severity of IRAEs. The publication date of the included studies was between 2015 and 2018. Additionally, two updated RCTs were included in this meta-analysis without duplicate counting of the sample (Hodi et al., 2016; Wolchok et al., 2017).

**Table 1:** The characteristics of included studies.

Study	Year	Study design	Histology	Age (years)	No. of patients (Male/Female)	Groups	NO. of Lost to Follow-up	CTCAE Version
Antonia et al., 2016	2016	Phase I/II RCT; Check Mate 032	SCLC	63 (57-68)	98 (61/37)	NIVO(3 mg/kg q2w)	0	4.0
				61 (56-65)	54 (32/22)	NIVO (3 mg/kg q3w) + IPI (1 mg/kg q3w)	0	
				66 (58-71)	61 (35/26)	NIVO (1 mg/kg q3w) + IPI (3 mg/kg q3w)	0	
Hellmann et al., 2017	2017	Phase III RCT; Check Mate 227	NSCLC	64 (median)	396 (273/123)	NIVO(3 mg/kg q2w)	5	4.0
				64 (median)	583 (391/192)	NIVO (3 mg/kg q2w) + IPI (1 mg/kg q6w)	7	
Larkin et al., 2015/ Wolchok et al., 2017	2015/2017	Phase III RCT; Check Mate 067	Melanoma	59 (25-90)	316 (202/144)	NIVO(3 mg/kg q2w)	3	4.0
				61 (18-89)	315 (202/113)	IPI (3 mg/kg q3w)	2	
				59 (18-88)	314 (206/108)	NIVO (1 mg/kg q3w) + IPI (3 mg/kg q3w)	3	
Postow et al., 2015/ Hodi et al., 2016	2015/2016	Phase III RCT; Check Mate 069	Melanoma	67 (31-80)	47 (32/15)	IPI (3 mg/kg q3w)	1	4.0
				64 (27-87)	95 (63/32)	NIVO (1 mg/kg q3w) + IPI (3 mg/kg q3w)	1	
Long et al., 2018	2018	Phase Ib RCT; KEYNOTE-029	Melanoma	63 (52-74)	25 (19/6)	NIVO (3 mg/kg q2w)	0	4.0
				59 (53-68)	35 (29/6)	NIVO (1 mg/kg q3w) + IPI (3 mg/kg q3w)	0	
D’angelo et al., 2018	2018	Phase III RCT; Alliance A091401	Sarcoma	56 (21-76)	43 (22/21)	NIVO (3 mg/kg q2w)	0	4.0
				57 (27-81)	42 (19/23)	NIVO (3 mg/kg q3w) + IPI (1 mg/kg q3w)	0	
Omuro et al., 2018	2018	Phase I RCT; CheckMate 143	Glioblastoma	58.5 (42-73)	10 (5/5)	NIVO (3 mg/kg q2w)	0	4.0
				60 (27-73)	20 (14/6)	NIVO (3 mg/kg q3w) + IPI (1 mg/kg q3w)	0	
				57 (37-68)	10 (6/4)	NIVO (1 mg/kg q3w) + IPI (3 mg/kg q3w)	0	
Sharma et al., 2019	2019	Phase I/II RCT; CheckMate 568	Urothelial Carcinoma	65.5 (31-85)	78 (54/24)	NIVO (3 mg/kg q2w)	0	4.0
				63.0 (39-83)	104 (81/23)	NIVO (3 mg/kg q2w) + IPI (1 mg/kg q2w)	0	
				64.0 (38-83)	92 (74/18)	NIVO (1 mg/kg q2w) + IPI (3 mg/kg q2w)	0	

NIVO, nivolumab; IPI, ipilimumab; No., number; CTCAE, Common Terminology Criteria for Adverse Events version; RCT, randomized controlled trials; NA, not available.

**Table 2** showed the methodological quality of the included studies. The randomization was reported in all the studies, and blinding of outcome assessment was reported in six studies. However, few studies described the allocation concealment and the blinding of participants during trial.

**Table 2:** Risk of bias in included studies.

**Study**	**Random Sequence Generation**	**Allocation Concealment**	**Blinding of Participants**	**Blinding of Outcome Assessment**	**Incomplete Outcome Data**	**Selective Reporting**	**Other Bias**
Antonia et al., 2016	Yes^a^	Unclear^b^	No^c^	Yes	Yes	Unclear	Unclear
Hellmann et al., 2017; Hellmann et al., 2018a; Hellmann et al., 2018b	Yes	Unclear	No	Yes	Yes	Unclear	Unclear
Larkin et al., 2015 /Wolchok et al., 2017	Yes	Unclear	Yes	Yes	Yes	Unclear	Unclear
Postow et al. 2015/ Hodi et al., 2016	Yes	Unclear	Yes	Yes	Yes	Unclear	Unclear
Long et al., 2018	Yes	Yes	Yes	Yes	Yes	Unclear	Unclear
D’angelo et al., 2018	Yes	Unclear	Unclear	Unclear	Yes	Unclear	Unclear
Omuro et al., 2018	Yes	Unclear	Unclear	Unclear	Yes	Unclear	Unclear
Sharma et al., 2019	Yes	Unclear	No	Yes	Yes	Unclear	Unclear

^a^Yes, low risk of bias; ^b^Unclear: unclear or unknown risk of bias; ^c^No: high risk of bias.

### Incidences of Organ-Specific IRAEs

Regarding any-grade organ-specific IRAEs associated with combination therapy, the most common adverse event was colitis (14.5%), followed by hypothyroidism (13.8%), hepatitis (10.4%), hypophysitis (10%), hyperthyroidism (9.3%), and pneumonitis (4.6%). While for 3-5 grade adverse events with monotherapy, the most common incidences were colitis (11.9%), hepatitis (3.7%), pneumonitis (1.7%), hypophysitis (1.1%), hypothyroidism (0.4%), and hyperthyroidism (0.4%).

### Outcomes of Meta-Analysis

The outcomes of meta-analysis were presented in **Table 3**, and the forest plots of meta-analysis were attached in **Supplementary Materials**.

**Table 3:** Meta-analysis of any-grade and 3-5 grade IRAEs between the ICI combination group and the monotherapy group.

Outcomes	Studies	Any Grade			3-5 Grade		
		Effect Estimate, RR (95% CI)	Overall Effect	Heterogeneity (*I* ^2^)	Effect Estimate, RR (95% CI)	Overall Effect	Heterogeneity (*I* ^2^)
Colitis	5	2.84 (1.42–5.65)	p = 0.003	I^2^ = 59%	3.71 (1.37–10.08)	p < 0.001	I^2^ = 55%
Pneumonitis	8	2.24 (1.52–3.32)	p < 0.001	I^2^ = 9%	1.96 (1.00–3.85)	p = 0.05	I^2^ = 0%
Hepatitis	4	2.16 (1.50–3.12)	p < 0.001	I^2^ = 24%	2.56 (1.27–5.16)	p = 0.009	I^2^ = 0%
Hypothyroidism	8	2.00 (1.61–2.48)	p < 0.001	I^2^ = 36%	2.34 (0.57–9.65)	p = 0.24	I^2^ = 0%
Hyperthyroidism	5	2.91 (1.98–4.29)	p < 0.001	I^2^ = 33%	6.98 (0.86–56.55)	p = 0.07	I^2^ = 0%
Hypophysitis	3	3.60 (1.31–9.86)	p = 0.01	I^2^ = 58%	0.45 (0.16–1.25)	p = 0.13	I^2^ = 11%

RR, risk ratio; CI, confidence interval.

#### Meta-Analysis of Any-Grade and 3-5 Grade Colitis

Five studies involving 1390 patients were included for meta-analysis (Antonia et al., 2016; Hodi et al., 2016; Wolchok et al., 2017; Long et al., 2018; Omuro et al., 2018). The incidences of any-grade colitis were 14.5% (85/587) vs 5.6% (45/803) in the combination vs monotherapy group; and 3-5 grade were 11.9% (70/587) vs 5.1% (41/803) in the combination vs monotherapy group. A random-effect model was used in the meta-analysis for significant heterogeneity among studies (*I* ^2^ > 50%). The results of the meta-analysis showed that patients treated with ICI combinations had significantly higher incidences of any-grade and 3-5 grade colitis when compared with the monotherapy group. The RR was 3.56 (95% CI, 1.56–8.12; *p* < 0.05) and 2.5 (95% CI, 1.62–3.86; *p* < 0.05) for any-grade and 3-5 grade colitis, respectively.

#### Meta-Analysis of Any-Grade and 3-5 Grade Pneumonitis

All the included studies involving 2716 patients reported any-grade and 3-5 grade pneumonitis. The incidences of any-grade pneumonitis were 4.6% (64/1401) vs 2.1% (27/1314) in the combination vs monotherapy group; and 3-5 grade were 1.7% (24/1401) vs 0.7% (9/1314) in the combination vs monotherapy group. A fixed-effect model was used in the meta-analysis for no significant heterogeneity among studies (*I* ^2^ < 50%). Meta-analysis showed significantly high incidences in any-grade and 3-5 grade pneumonitis in the ICI combination group. The RR was 2.31 (95% CI, 1.54–3.45; *p* < 0.05) and 1.99 (95% CI, 1.00–3.93; *p* = 0.05) for any-grade and 3-5 grade pneumonitis, respectively.

#### Meta-Analysis of Any-Grade and 3-5 Grade Hepatitis

Four studies involving 1441 patients were included for meta-analysis (Hodi et al., 2016; Hellmann et al., 2018b; Long et al., 2018; Sharma et al., 2019). The incidences of any-grade hepatitis were 10.4% (94/901) vs 7.1% (24/340) in the combination vs monotherapy group; and 3-5 grade were 3.7% (33/901) vs 2.1% (7/340) in the combination vs monotherapy group. No significant heterogeneity was found among studies (*I* ^2^ < 50%). Meta-analysis demonstrated that, the ICI combination group had significantly higher any-grade and 3-5 grade hepatitis than the monotherapy group. The RR was 2.54 (95% CI, 1.65–3.91; *p* < 0.05) and 2.70 (95% CI, 1.29–5.63; *p* < 0.05) for any-grade and 3-5 grade hepatitis, respectively.

#### Meta-Analysis of Any-Grade and 3-5 Grade Hypothyroidism

All studies reported the incidence of hypothyroidism. The incidences of any-grade hypothyroidism were 13.8% (194/1401) vs 7.2% (95/1315) in the combination vs monotherapy group; and 3-5 grade were 0.4% (5/1401)vs 0.1% (1/1315) in the combination vs monotherapy group. There was no significant heterogeneity among studies (*I* ^2^ < 50%). Compared with the monotherapy group, the combination group showed significant higher risks in any-grade hypothyroidism, and the RR was 2.17 (95% CI, 1.71–2.76; *p* < 0.05). However, no difference was found in 3-5 grade hypothyroidism (RR, 2.36; 95% CI, 0.55–10.13; *p* = 0.25).

#### Meta-Analysis of Any-Grade and 3-5 Grade Hyperthyroidism

Five studies involving 1524 patients were included for meta-analysis (Antonia et al., 2016; Wolchok et al., 2017; Long et al., 2018; Omuro et al., 2018; Sharma et al., 2019). The incidences of any-grade hyperthyroidism were 9.3% (64/689) vs 3.0% (25/835) in the combination vs monotherapy group; and 3-5 grade were 0.4% (3/689) vs 0% (0/835) in the combination vs monotherapy group. The heterogeneity was not significant among studies (*I* ^2^ < 50%). Meta-analysis showed that patients receiving ICI combination therapy had significantly higher risk in any-grade hyperthyroidism than those receiving monotherapy, and the RR was 3.13 (95% CI, 2.08–4.70; *p* < 0.05), but no difference was found in 3-5 grade hyperthyroidism (RR, 7.05; 95% CI, 0.86–57.43; *p* = 0.07).

#### Meta-Analysis of Any-Grade and 3-5 Grade Hypophysitis

Three studies involving 1137 patients reported the incidence of hypophysitis (Hodi et al., 2016; Wolchok et al., 2017; Long et al., 2018). The incidences of any-grade hypophysitis were 10.0% (44/442) vs 2.4% (17/695) in the combination vs monotherapy group; and 3-5 grade were 1.1% (5/442) vs 1.6% (11/695) in the combination vs monotherapy group. No significant heterogeneity was found among studies (*I* ^2^ < 50%). Meta-analysis showed that the combination group had significant high risks in any-grade hypophysitis, and the RR was 3.54 (95% CI, 2.07–6.07; *p* < 0.05). No difference was found in 3-5 grade hypophysitis (RR, 0.45; 95% CI, 0.16–1.23; *p* = 0.12).

### Meta-Analysis of Total Treatment-Related Adverse Events

A total of 2,716 patients were included in 10 studies with 1315 in the monotherapy group (nivolumab, 958; ipilimumab, 357) and 1,401 in the combination group (nivolumab and ipilimumab). A random-effect model was used for the outcome of total 3-5 grade adverse events due to significant heterogeneity among studies (*I* ^2^ > 50%). When compared with the monotherapy group, meta-analysis showed that patients in the ICI combination group had significantly higher risks for total treatment-related adverse events in any and 3-5 grade. The RR was 1.68 (95% CI, 1.35–2.08; *p* < 0.05) and 2.99 (95% CI, 2.00–4.46; *p* < 0.05) for total any-grade and 3-5 grade hepatitis, respectively.

### Subgroups Analysis

#### Different Types of Tumors

As for the insufficient number of included studies on lung cancer, glioblastoma, urothelial carcinoma, and sarcoma, only one subgroup analysis was performed on melanoma. Meta-analysis showed that, the combination therapy significantly increased the risks of total 3-5 grade organ-specific IRAEs (RR, 1.70; 95% CI, 1.25–2.30; *p* < 0.05) in melanoma patients, but no difference was found in the incidences of total any-grade IRAEs between both groups (RR, 1; 95% CI, 0.99–1.01; *p* = 0.77).

#### Different Drug Doses

The incidences of IRAEs based on drugs (nivolumab alone, ipilimumab alone, and nivolumab plus ipilimumab) were summarized in **Table 4**. In the combination group, three studies included two different doses of drug combinations: nivolumab 3 mg/kg plus ipilimumab 1 mg/kg (N3I1), or nivolumab 1 mg/kg plus ipilimumab 3 mg/kg (N1I3) (Antonia et al., 2016; Omuro et al., 2018; Sharma et al., 2019). The subgroups analysis showed that there was no difference in the incidence of total any-grade organ-specific IRAEs between N3I1 and N1I3 groups (RR, 0.99; 95% CI, 0.89–1.09; *p* = 0.84), but the incidence of the total 3-5 grade IRAEs was significantly higher in the N1I3 group (RR, 1.70; 95% CI, 1.25–2.30; *p* < 0.05).

**Table 4:** Incidence of the organ-specific IRAEs by drug (%).

Drugs	Colitis		Pneumonitis		Hepatitis		Hypothyroidism		Hyperthyroidism		Hypophysitis	
	Any grade	Grade 3-5	Any grade	Grade 3-5	Any grade	Grade 3-5	Any grade	Grade 3-5	Any grade	Grade 3-5	Any grade	Grade 3-5
Nivolumab + Ipilimumab^a^	14.5 (85/587)	11.9 (70/587)	4.6 (64/1401)	1.7 (24/1401)	10.4 (94/901)	3.7 (33/901)	13.8 (194/1401)	0.4 (5/1401)	9.3 (64/689)	0.4 (3/689)	10.0 (44/442)	1.1 (5/442)
Nivolumab	1.6 (7/446)	0.7 (3/446)	2.3 (22/957)	0.8 (8/957)	4.9 (24/294)	1.4 (7/294)	7.8 (75/958)	0.1 (1/958)	4.0 (21/524)	0 (0/524)	0.6 (2/338)	1.5 (5/338)
Ipilimumab	10.6 (38/357)	10.6 (38/357)	1.4 (5/357)	0.3 (1/357)	0 (0/46)	0 (0/46)	5.6 (20/357)	0 (0/357)	1.3 (4/311)	0 (0/311)	4.2 (15/357)	1.7 (6/357)

^a^Includes both two different doses of drug combinations: Nivolumab 3 mg/kg plus Ipilimumab 1 mg/kg (N3I1), nivolumab 1 mg/kg plus ipilimumab 3 mg/kg.

### Publication Bias

The funnel plot was used to explore the potential publication bias. All the included studies showed a symmetric distribution on the funnel plots. No significant publication bias was found in this meta-analysis.

## Discussion

In this study, 10 literatures involving 8 RCTs with 2716 patients were included for meta-analysis. The most important finding of this study is that the use of ICI combination (nivolumab and ipilimumab) significantly increased the risks in any-grade IRAEs in colitis, pneumonitis, hepatitis, hypothyroidism, hyperthyroidism, and hypophysitis, as well as the 3-5 grade IRAEs in colitis, pneumonitis, and hepatitis.

The rapid development of ICIs has dramatically changed the therapeutic options in numerous cancers. Compared with ICI monotherapy, ICI combination therapy has become a more popular therapeutic way for its superior clinical efficacy. However, few studies are focused on the organ-specific IRAEs. Although a previous meta-analysis by Wang et al. (2018) had assessed the toxic effects caused by ICIs, the authors only reported the mortality related to the ICI toxicity (122 deaths in 19,217 patients). Nevertheless, for a prompt recognition and management of adverse events, we should not only know the epidemiology regarding the fatal events, but also for moderate and severe adverse effects (Martins et al., 2019). Therefore, we designed this study to compare the risks of any-grade and 3-5 grade adverse effects associated with ICI combination therapy with monotherapy.

In our study, colitis and hepatitis were included as ICI-induced gastrointestinal and hepatic injury. The most frequent IRAE associated with combination therapy was colitis (any grade, 14.5%; 3-5 grade: 11.9%), with a significantly higher incidence than that in the monotherapy group (any grade: 5.6%; 3-5 grade: 3.5%). Hepatitis induced by ICI was less frequent compared to colitis, occurring in approximately 10.4% of patients receiving ICI combination therapy, with 3.7% above grade 3. Meta-analysis showed that ICI combination therapy significantly increased risks of colitis and hepatitis than ICI monotherapy. Of note, the increased colitis in the combination therapy group might be mainly contributed to the use of anti-CTLA-4 drugs. Earlier studies demonstrated a higher incidence of gastrointestinal adverse events associated with CTLA-4 inhibitors alone compared with anti-PD-1 therapy. In a large-sample phase-3 study with 945 patients, Larkin et al. (2015) compared the safety of nivolumab alone, ipilimumab alone, and nivolumab plus ipilimumab. The results showed that colitis of any grade occurred in 0.6% of the patients in the nivolumab group, 7.7% of those in the ipilimumab group, and 8.3% of those in the nivolumab-plus-ipilimumab group, respectively. This result was consistent with our subgroup analysis, which showed that patients receiving ipilimumab alone (10.6%) more likely experienced any-grade and serious colitis than those who received nivolumab alone (1.6%). Currently, the related pathogenesis of colitis initiated by ipilimumab still remains unclear. Histopathologic features might be related with an increase in intraepithelial lymphocytes for CTLA-4 inhibitors (Gupta et al., 2015; Weidner et al., 2015).

For the organ-specific IRAEs in thyroid dysfunction, we included the outcomes of hypothyroidism and hyperthyroidism in this study. Meta-analysis showed that the combination group had significant high risks in any-grade hypothyroidism and hyperthyroidism compared with the monotherapy group. In terms of 3-5 grade adverse events, prior studies demonstrated that ICI therapy rarely resulted in serious thyroid dysfunction (Morganstein et al., 2017; Zhang et al., 2018). This study revealed that only 3 (0.4%, 3/1401) patients had serious hypothyroidism in combination therapy group, while 1 (0.1%, 1/958) and 5 (0.4%, 5/958) patients had serious hyperthyroidism in nivolumab and combination groups, respectively. Meta-analysis showed no differences between the monotherapy and combination groups. Hypophysitis was regarded as the most frequent endocrine dysfunction caused by ICI therapy. Notably, we found that the variation tendency of hypophysitis was similar with that of thyroid dysfunction, which showed that the combination group had statistically higher incidence in all-grade events, but no difference in serious events between two groups. A possible explanation was that the thyrotropin hormone was affected by hypophysitis, thus resulting in thyroid disorders (Barroso-Sousa et al., 2018; Zhang et al., 2018).

Pneumonitis was a relatively rare adverse event during checkpoint inhibition therapy, which appeared more prevalent in lung cancer patients (Spain et al., 2016). Low-grade pneumonitis was commonly manageable with treatment discontinuation, but serious pneumonitis is potentially life-threatening (Martins et al., 2019; Rahouma et al., 2019). In this study, meta-analysis revealed that ICI combination therapy was associated with a significantly higher risk of pneumonitis compared with monotherapy. Subgroup analysis revealed that the rates of all-grade pneumonitis were 4.6%, 2.3% and 1.4% in patients receiving nivolumab plus ipilimumab, nivolumab alone, and ipilimumab alone, respectively. Interestingly, unlike colitis, pneumonitis was more frequent among patients receiving anti-PD-1/PD-L1 therapies as opposed to those receiving anti-CTLA4 therapies (Martins et al., 2019). Analysis based on the drug types also showed that patients receiving anti-PD-1 inhibitors (nivolumab) experienced more high-grade pneumonitis than those receiving anti-CTLA4 inhibitors (ipilimumab), and the incidences were 0.8% and 0.3%, respectively. Moreover, 1 (1.1%) case and 3 cases (2.3%) of pneumonitis-related death associated with nivolumab were reported by Postow et al. (2015) and Gettinger et al. (2015), respectively. Therefore, despite a relatively low incidence of pneumonitis, this adverse effect should be closely followed up by clinicians, in particular when anti-PD-1/PD-L1 inhibitors are being used.

For the subgroup analysis, we found that the organ-specific IRAEs appeared to be drug- and dose-dependent. Regarding the drug dependent, the risks of colitis and hypophysitis appeared to be more related to the CTLA-4 antibodies (ipilimumab); the pneumonitis and hepatitis appeared to be more related to the PD-1 antibodies (nivolumab). Regarding the dose dependent, we compared two different doses in drug combinations (nivolumab 3 mg/kg pus ipilimumab 1 mg/kg versus nivolumab 1 mg/kg plus ipilimumab 3 mg/kg). The result showed that nivolumab 3 mg/kg plus ipilimumab 1 mg/kg significantly increased the total 3-5 grade IRAEs.

## Study Strengths and Limitations

The most important strength of this study is that all the included studies are RCTs with detailed registration information in ClinicalTrials.gov. Meanwhile, there are also several limitations in this study. First, the number of the included RCTs was small (10 studies involving 8 RCTs and 2,716 patients), which limited us to perform subgroup analysis. Further high-quality RCTs with large sample sizes are needed to verify our conclusion. Second, some definitions of adverse events were not uniform. For example, immune-related hepatitis was reported as hepatitis or increased aspartate transaminase/alanine transaminase, and immune-mediated colitis was reported as colitis or diarrhea, which might lead to incomplete data collection. This should be standardized in the future study. Third, patients with various cancers were included, which might have bias in the incidence of some adverse effects. For example, lung cancer was related with a high risk of developing pneumonitis from previous lung disease, radiotherapy, and smoking history. Subgroup analysis was not done based on different types of cancers due to the insufficient studies on lung cancer, sarcoma, glioblastoma, and urothelial carcinoma. Fourth, heterogeneity was found in the outcomes of colitis and total adverse events among the included studies. The heterogeneity might have resulted from the differences of cancer types, follow-up time, drug dose, and so on.

## Conclusions

This meta-analysis demonstrated that, compared with ICI monotherapy, combination therapy with ICI drugs significantly increased the risk of organ-specific IRAEs in colitis, hypothyroidism, hepatitis, hypophysitis, hyperthyroidism, and pneumonitis. The incidence and severity of organ-specific IRAEs were drug- and dose-independent. Although the incidence of high-grade organ-specific IRAEs was relatively low, clinicians should be aware of these adverse effects so that patients can be promptly managed.

## Author Contributions

Study concept and design: LD, YT, FS. Data extraction and analysis: LD, NW, KZ, YQ. Manuscript writing: LD, YT, NW, KZ, FS. Revision of the manuscript: LD, YT, KZ, YQ, NW, YL. Technical or material support: YQ, NW, FS.

## Conflict of Interest

The authors declare that the research was conducted in the absence of any commercial or financial relationships that could be construed as a potential conflict of interest.
