# Supplementary figures and images for: Organ-Specific Immune-Related Adverse Events Associated With Immune Checkpoint Inhibitor Monotherapy Versus Combination Therapy in Cancer: A Meta-Analysis of Randomized Controlled Trials
Source: Front Pharmacol. 2020 Jan 30;10:1671. doi: 10.3389/fphar.2019.01671 (PMC7002539; doi:10.3389/fphar.2019.01671)

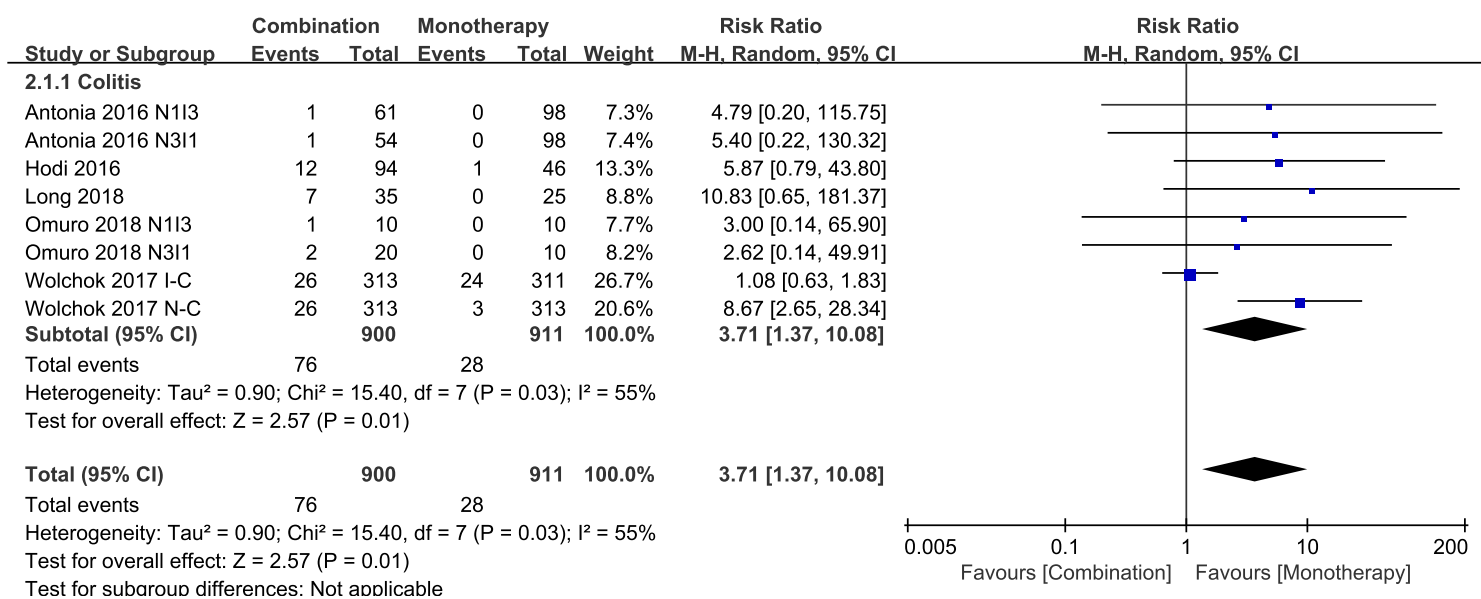

Supplement: Supplementary file 1 [file DataSheet_1.zip › Supplemental Files/Meta-analysis of 3-5 grade Colitis.pdf]

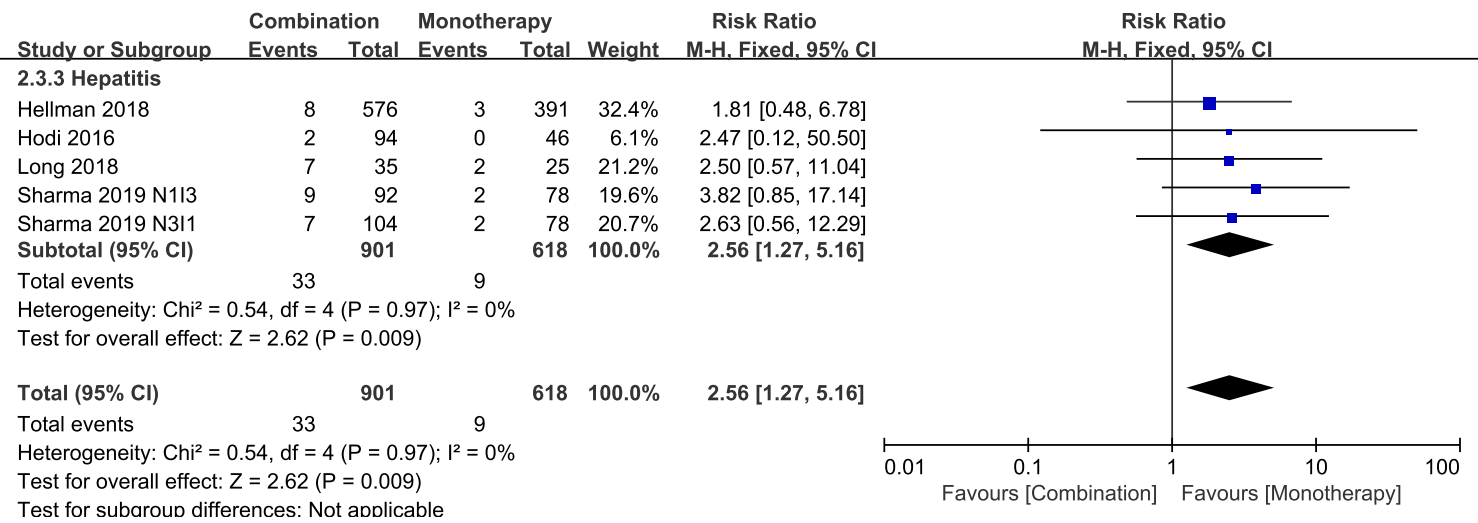

Supplement: Supplementary file 1 [file DataSheet_1.zip › Supplemental Files/Meta-analysis of 3-5 grade Hepatitis.pdf]

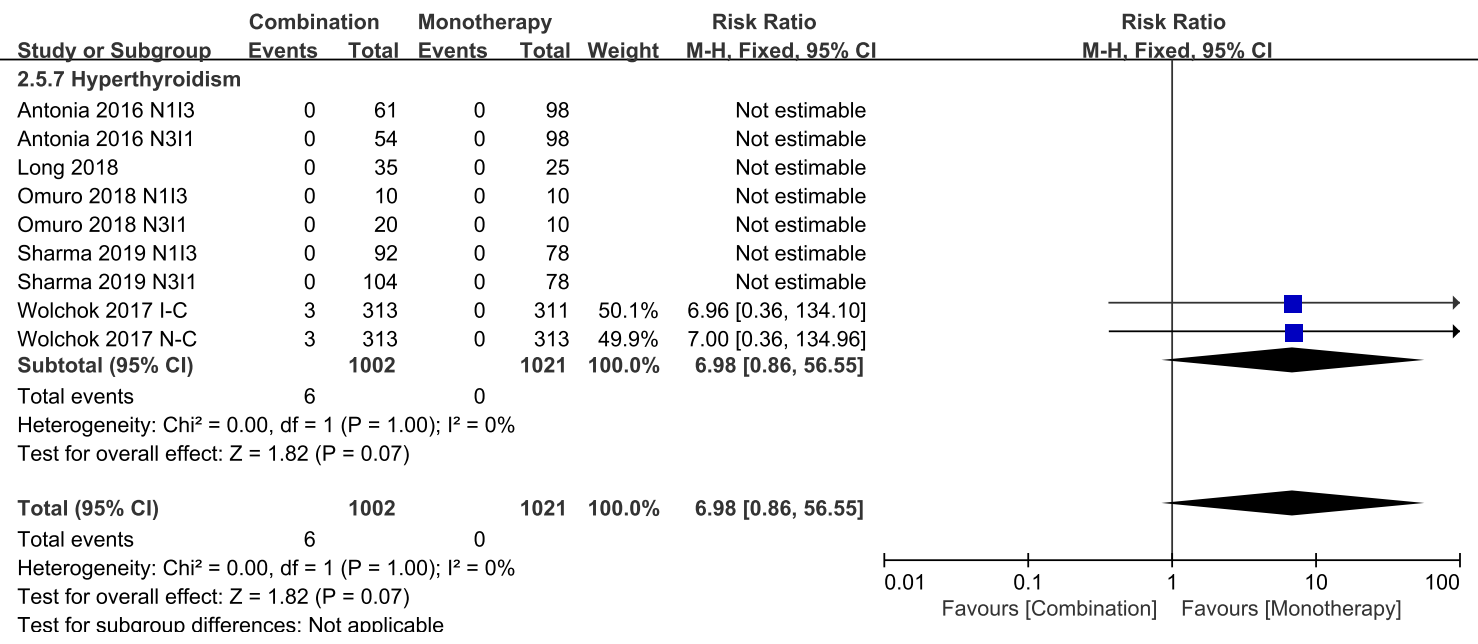

Supplement: Supplementary file 1 [file DataSheet_1.zip › Supplemental Files/Meta-analysis of 3-5 grade Hyperthyroidism.pdf]

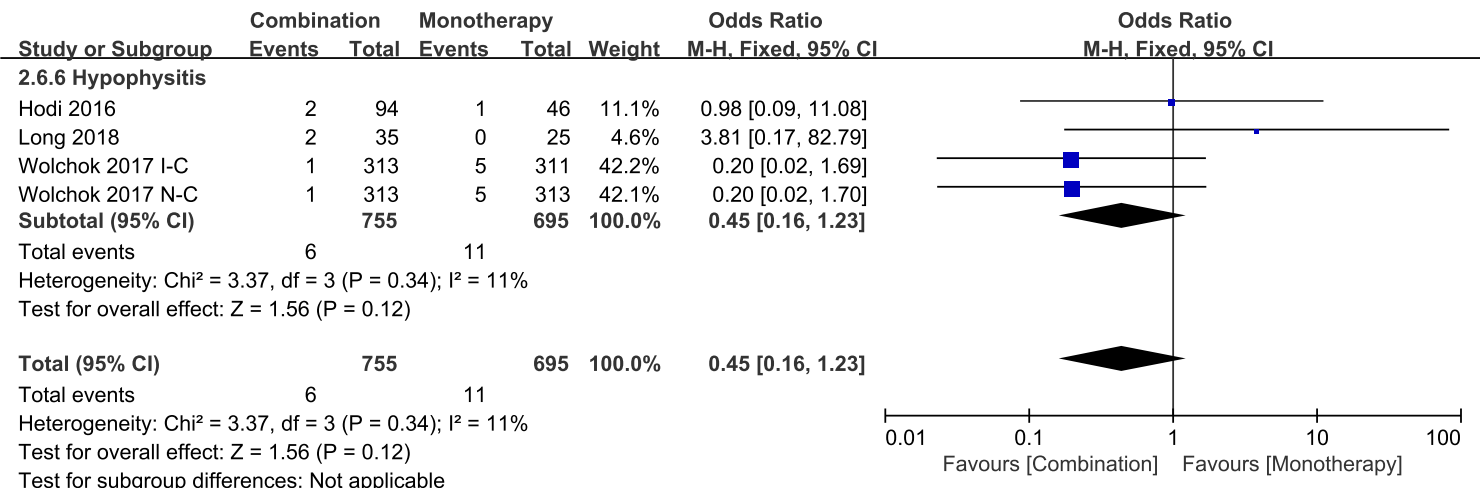

Supplement: Supplementary file 1 [file DataSheet_1.zip › Supplemental Files/Meta-analysis of 3-5 grade Hypophysitis.pdf]

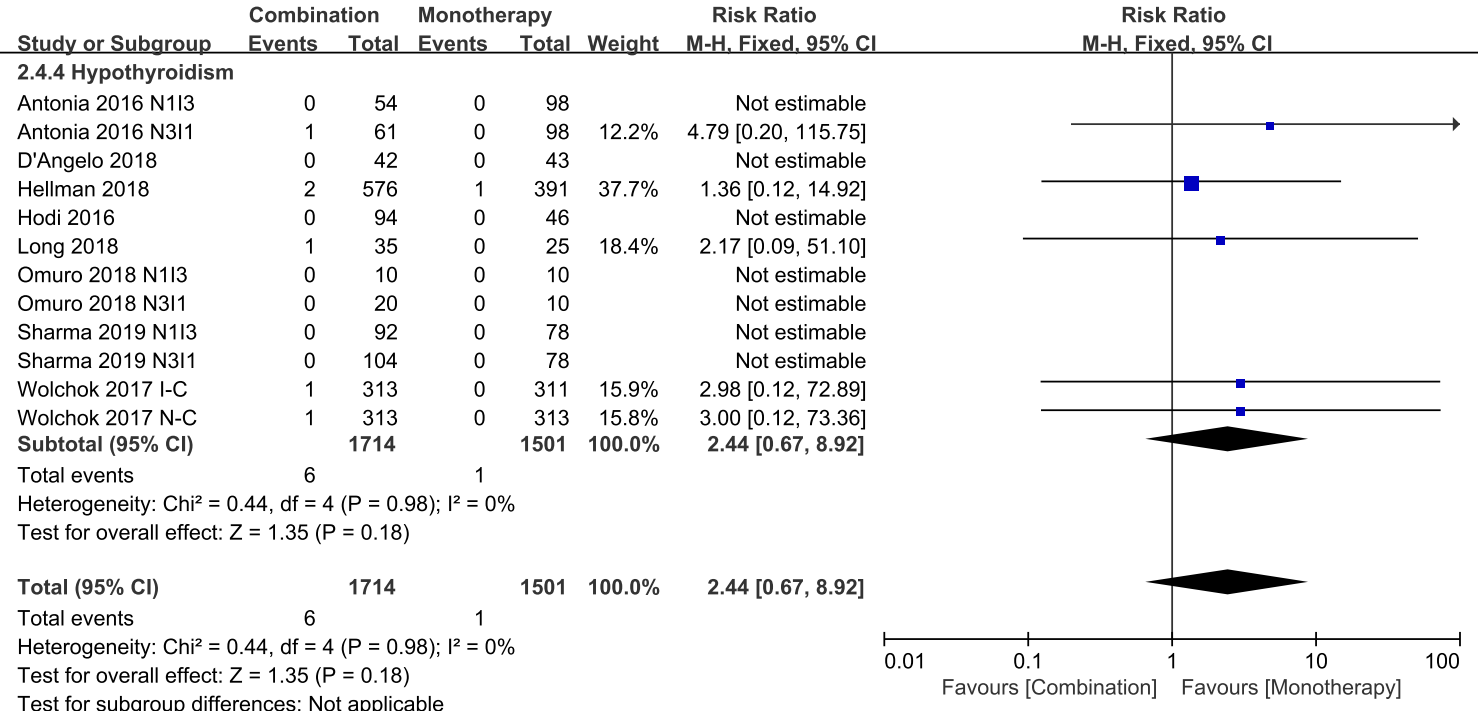

Supplement: Supplementary file 1 [file DataSheet_1.zip › Supplemental Files/Meta-analysis of 3-5 grade Hypothyroidism.pdf]

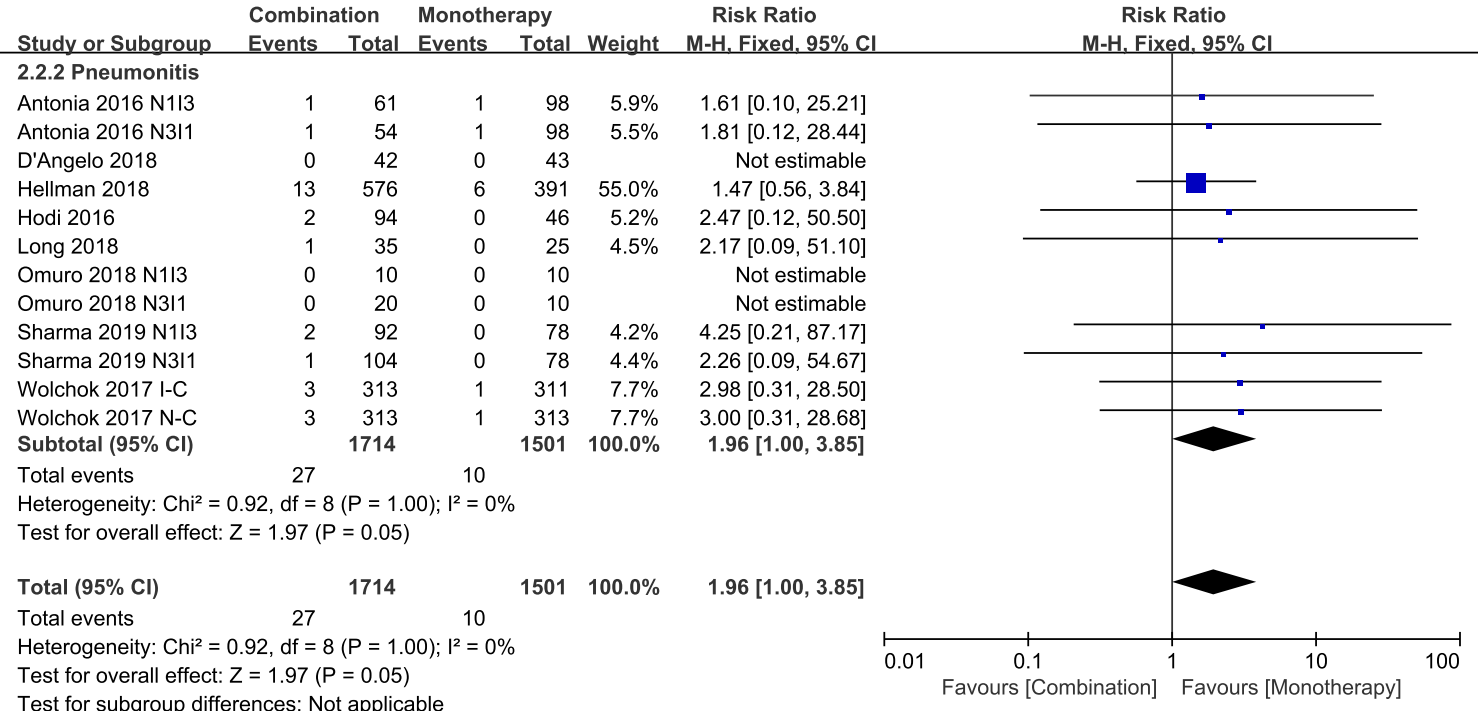

Supplement: Supplementary file 1 [file DataSheet_1.zip › Supplemental Files/Meta-analysis of 3-5 grade Pneumonitis.pdf]

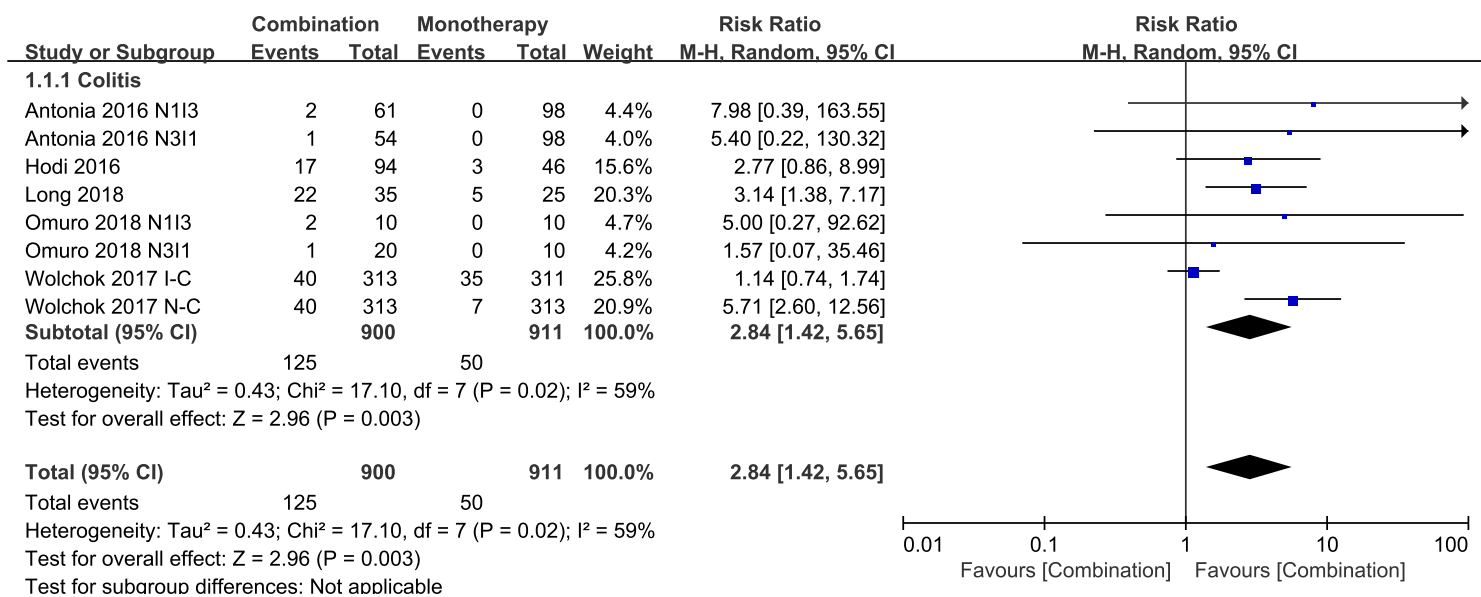

Supplement: Supplementary file 1 [file DataSheet_1.zip › Supplemental Files/Meta-analysis of Any-grade Colitis.pdf]

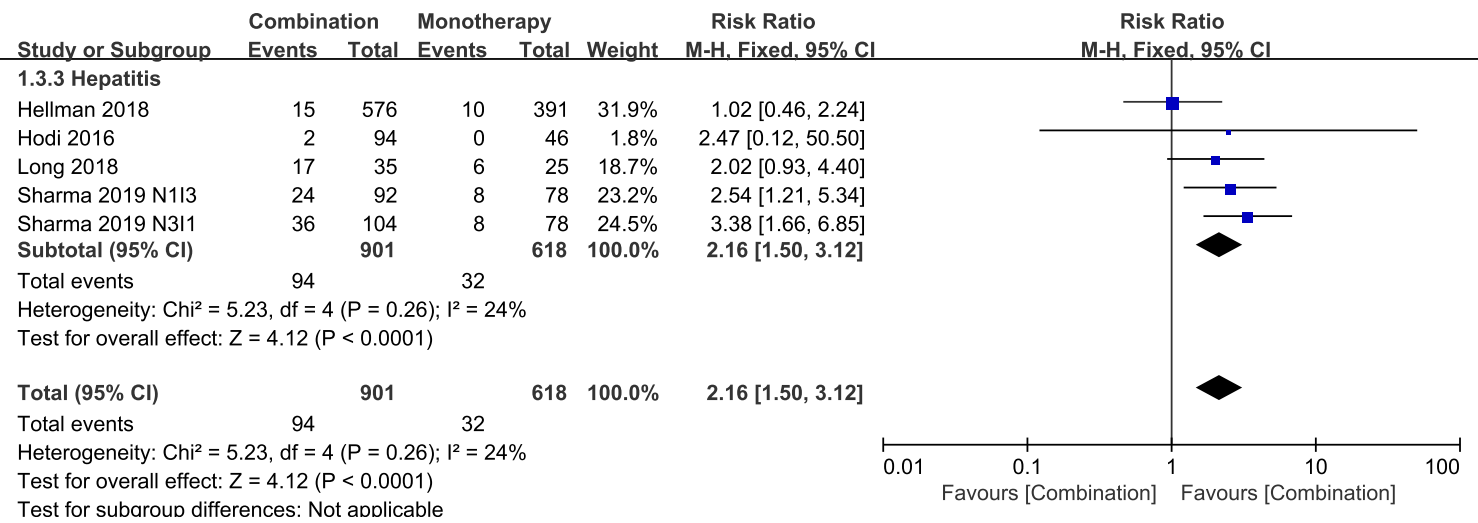

Supplement: Supplementary file 1 [file DataSheet_1.zip › Supplemental Files/Meta-analysis of Any-grade Hepatitis.pdf]

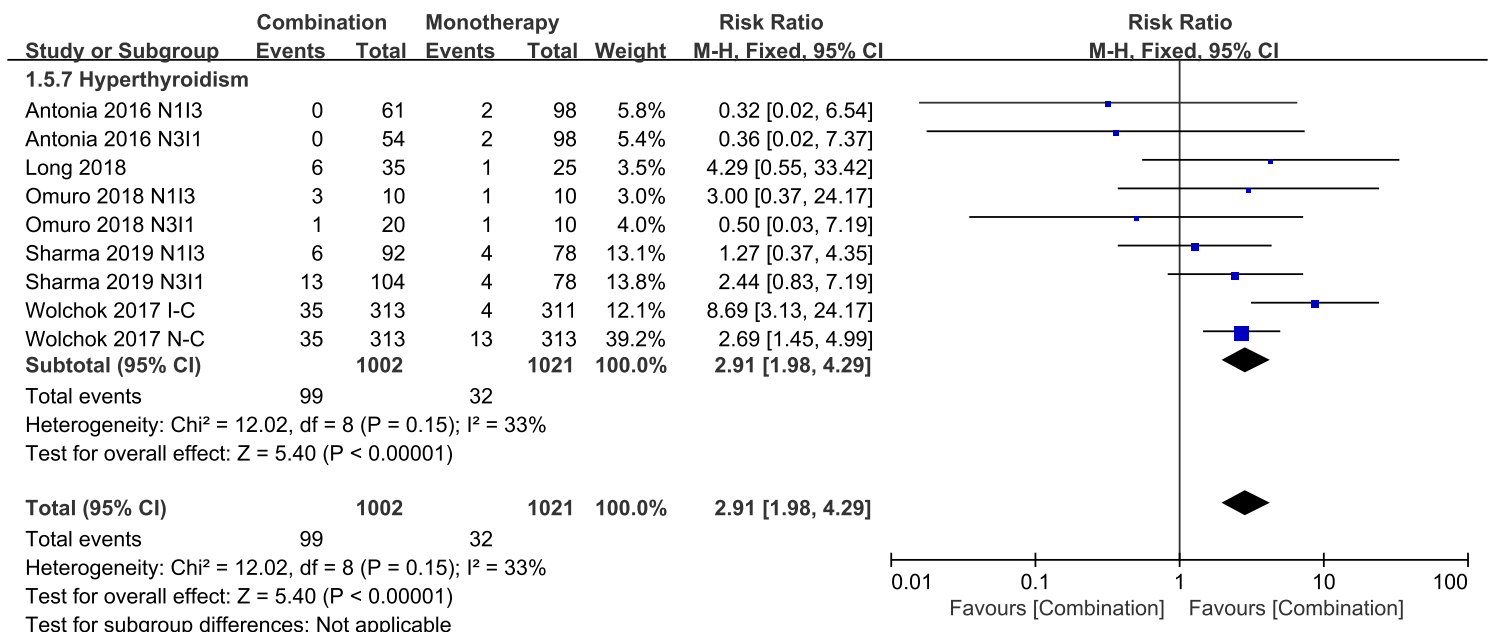

Supplement: Supplementary file 1 [file DataSheet_1.zip › Supplemental Files/Meta-analysis of Any-grade Hyperthyroidism.pdf]

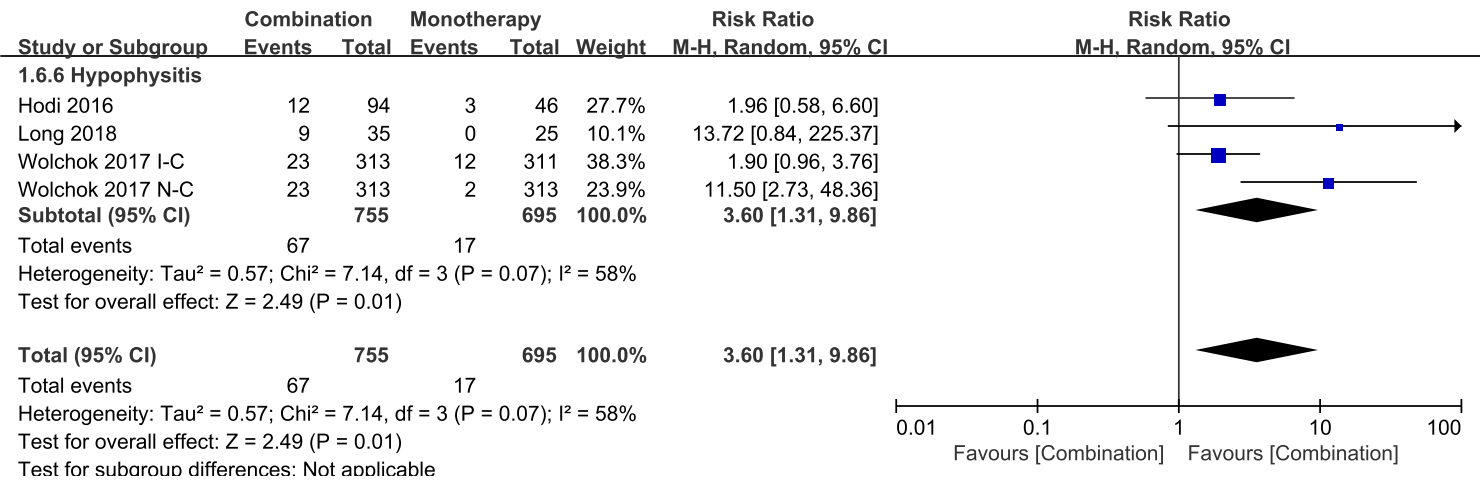

Supplement: Supplementary file 1 [file DataSheet_1.zip › Supplemental Files/Meta-analysis of Any-grade Hypophysitis.pdf]

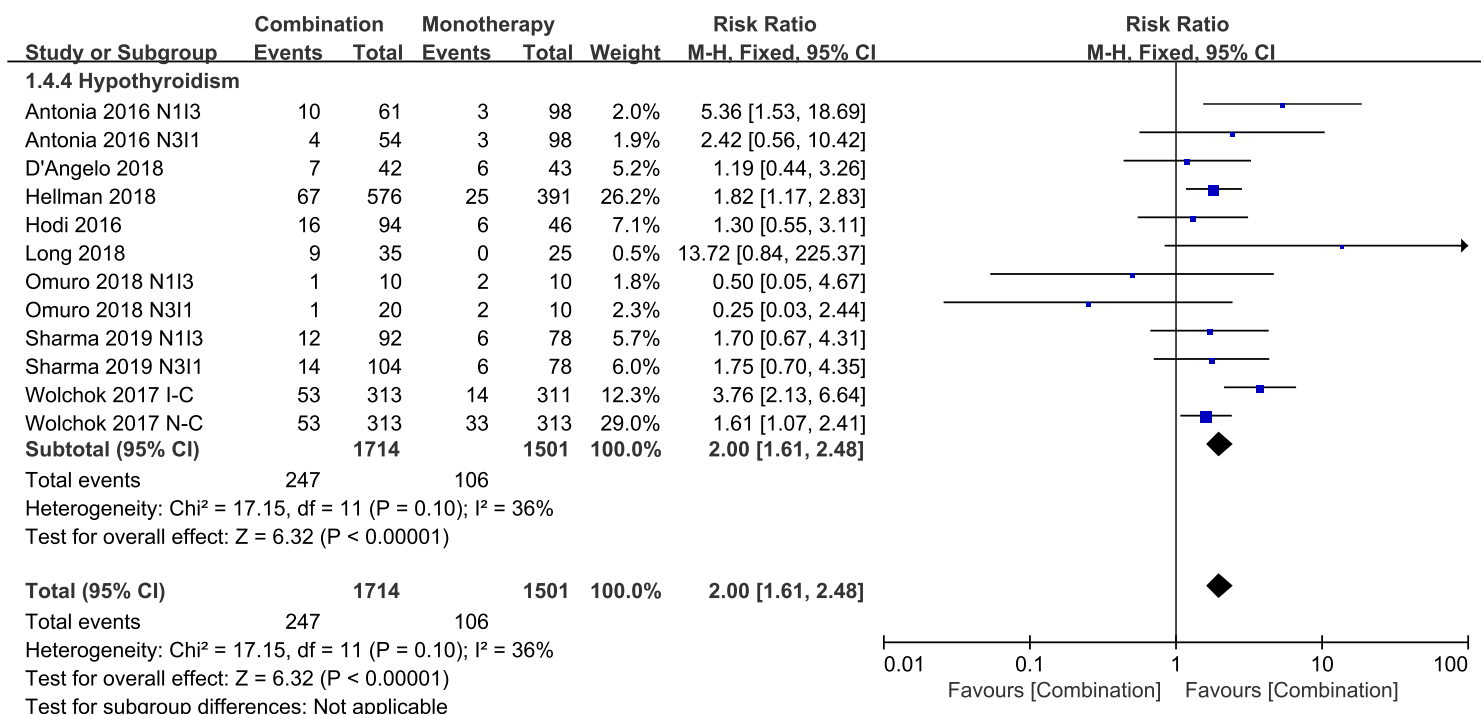

Supplement: Supplementary file 1 [file DataSheet_1.zip › Supplemental Files/Meta-analysis of Any-grade Hypothyroidism.pdf]

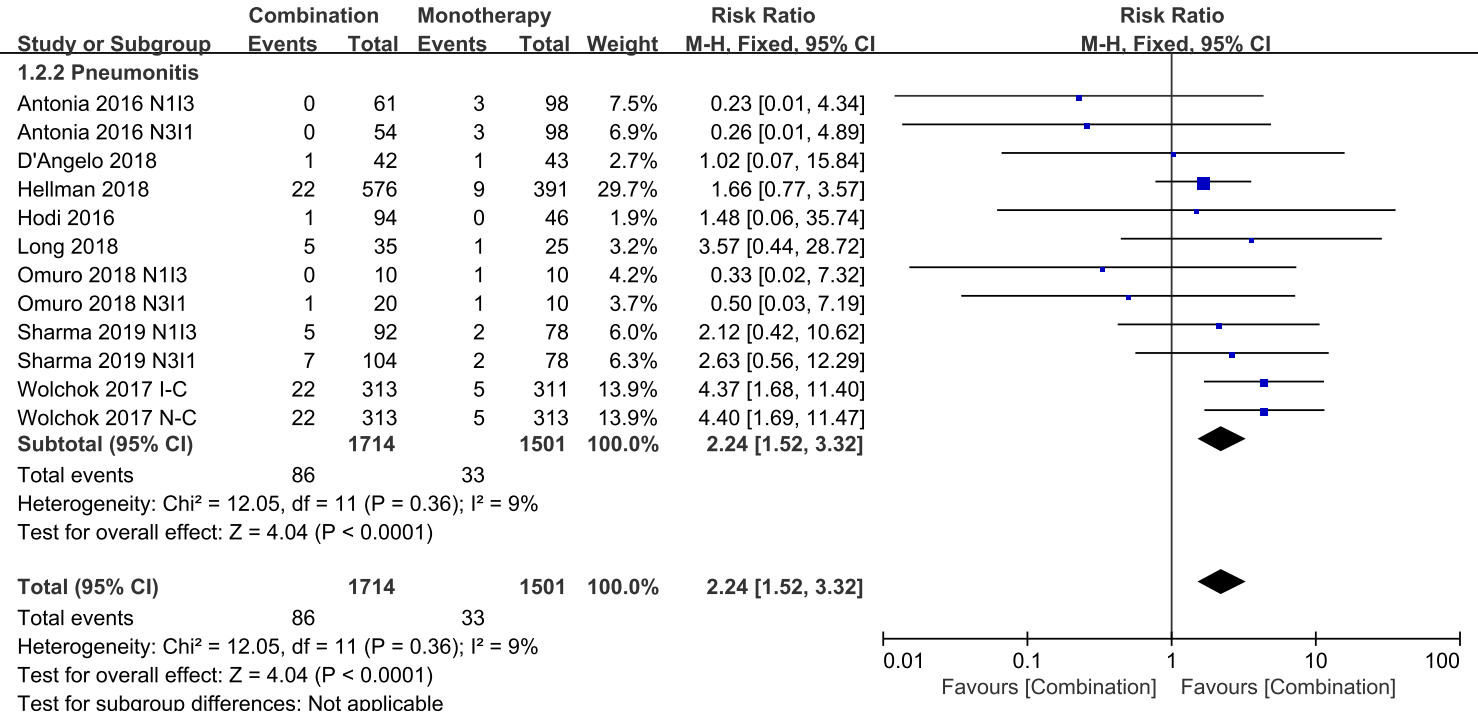

Supplement: Supplementary file 1 [file DataSheet_1.zip › Supplemental Files/Meta-analysis of Any-grade Pneumonitis.pdf]
